# Towards a Cognitive Radar: Canada’s Third-Generation High Frequency Surface Wave Radar (HFSWR) for Surveillance of the 200 Nautical Mile Exclusive Economic Zone

**DOI:** 10.3390/s17071588

**Published:** 2017-07-07

**Authors:** Anthony Ponsford, Rick McKerracher, Zhen Ding, Peter Moo, Derek Yee

**Affiliations:** 1 Former Employee of Raytheon Canada Limited, 400 Phillip Street, Waterloo, ON N2L 6R7, Canada; AM.Ponsford@gmail.com (A.P.); yeed.8888@gmail.com (D.Y.); 2 Raytheon Canada Limited, 400 Phillip Street, Waterloo, ON N2L 6R7, Canada; Rick_McKerracher@raytheon.com; 3 Radar Sensing and Exploitation Section, Defence Research and Development Canada; Ottawa, ON K1A 0Z4, Canada; Zhen.Ding@drdc-rddc.gc.ca

**Keywords:** over-the-horizon radar, HFSWR, cognitive, sense-and-adapt, dynamic spectrum management, dynamic spectrum access, maritime domain awareness, maritime surveillance, MIMO

## Abstract

Canada’s third-generation HFSWR forms the foundation of a maritime domain awareness system that provides enforcement agencies with real-time persistent surveillance out to and beyond the 200 nautical mile exclusive economic zone (EEZ). Cognitive sense-and-adapt technology and dynamic spectrum management ensures robust and resilient operation in the highly congested High Frequency (HF) band. Dynamic spectrum access enables the system to simultaneously operate on two frequencies on a non-interference and non-protected basis, without impacting other spectrum users. Sense-and-adapt technologies ensure that the system instantaneously switches to a new vacant channel on the detection of another user or unwanted jamming signal. Adaptive signal processing techniques mitigate against electrical noise, interference and clutter. Sense-and-adapt techniques applied at the detector and tracker stages maximize the probability of track initiation whilst minimizing the probability of false or otherwise erroneous track data.

## 1. Introduction to Cognitive Radar

Early radars were cognitive in the sense that they generally required a person-in-the-loop to optimize performance based on observed data. The goal of modern radar developments has been to replace the person-in-the-loop. This has led to the pursuance of various techniques that adapt the radar and tracking parameters to the mission and sensed conditions. Cognition takes this one step further, adding more and more intelligence to the optimization process. This intelligence may be internally learned or gained from trusted third-party sources. 

**Figure 1:**
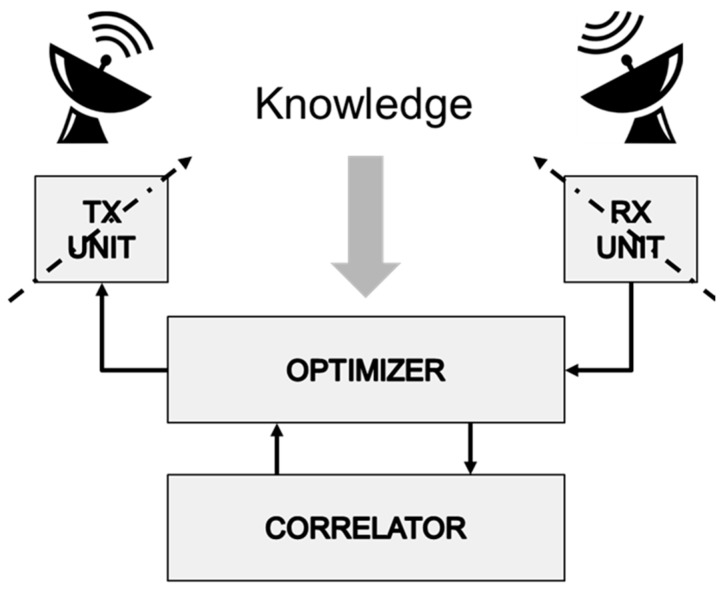
Basic components of a cognitive radar.

Figure 1 illustrates the core components of a cognitive radar system. The optimizer senses the environment and adapts the transmitter, receiver and processing parameters with the objective of maximizing the wanted signal, whilst simultaneously minimizing the unwanted signal. The correlator isolates and associates time sequential responses with common attributes to maximize the probability of track initiation, whilst minimizing the probability of a missed track or a false track. 

Both the adaptive radar and cognitive radar utilize feedback from the receiver to the transmitter. 

However, a cognitive radar also incorporates memory and learning. Intelligence and knowledge can be generated by ingesting data from other trusted sources. 

Cognition, as defined in [1], utilizes intelligent signal processing, which builds on learning through interactions of the radar with the surrounding environment, as well as feedback from the receiver to the transmitter, which is one of the facilitators of intelligence. A cognitive radar uses its own data, as well as trusted third-party knowledge, to extend the radar’s capability. For example, if a target is being observed by other means or is known to be of low-value for the mission, then resources can be diverted to higher-priority targets. Alternatively, data related to weather conditions, or other environmental data such as wind speed and direction or ambient temperature data, can be used to aid the optimization of the radar.

A cognitive radar utilizes the principle of dynamic spectrum access (DSA), which was developed for cognitive radio. DSA is a spectrum-sharing scheme that allows radiating systems to operate as secondary users on a non-interference and non-protected basis (NIB and NPB). DSA enables significant improvement in the efficient use of the rf spectrum, and maximizes the number of users of the spectrum compared to traditional fixed-spectrum access. DSA also opens up the feasibility of spectrum sharing between radar and communication users.

**Figure 2:**
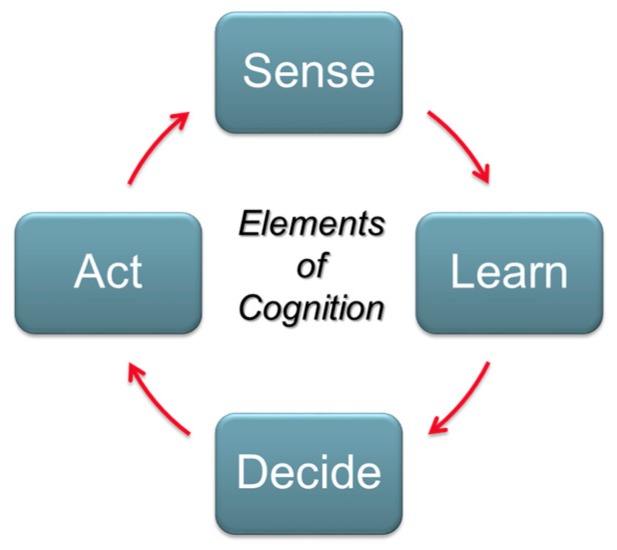
The cognitive loop, where “Sense” relates to the collecting and processing of radar data, “Learn” is the understanding of the environment and may include information from trusted sources, “Decide” is the determination of the optimization of the radar, and “Act” is the modification of the radar parameters to meet the mission objectives in the known environment.

Cognitive techniques allow a radar to adapt to a changing threat and clutter environment by optimizing performance. Previous work on cognitive systems and waveform design was presented in [2,3,4,5,6]. A pictorial representation of the cognitive cycle is illustrated in Figure 2, where “Sense” relates to the collecting and processing of data, and “Learn” is the understanding of the environment based on the sensed data relative to the mission profile. This data may include information from independent trusted sources to aid in the detection and tracking process. “Decide” is the determining of the optimization of the radar algorithms, and “Act” is the modification of the radar parameters (waveform, detector, tracker, etc.) to meet the mission objective. The primary objective of the cognitive system is to detect and track targets via the effective allocation of radar resources, with a secondary objective to confirm the identification of known targets. It can be noted that the cognitive cycle is similar in context to the standard OODA decision cycle: observe, orient, decide, and act. 

## 2. Canada’s Third-Generation HFSWR

Canada’s third-generation HFSWR system has been developed by Raytheon Canada in collaboration with Defence Research and Development Canada, based on a cognitive sense-and-adapt architecture [7]. The objective was to utilize knowledge obtained either by sensing the local environment or from trusted third-party sources to maximize the probability of track initiation, whilst minimizing the probability of false or otherwise erroneous tracks. 

**Figure 3:**
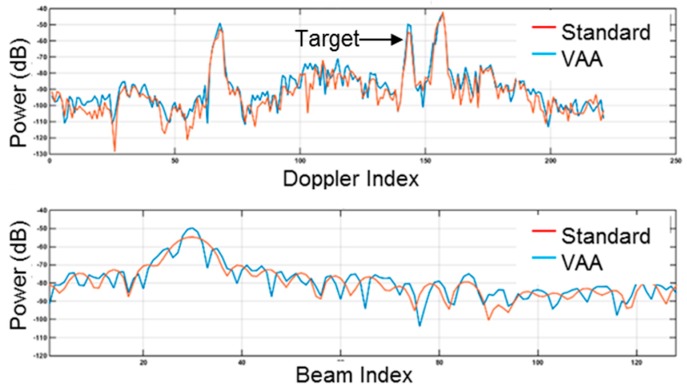
Comparison of the performance of the virtual aperture array technique relative to standard showing an approximately 3 dB improvement in the target-to-clutter ratio and a reduction of the azimuth beam width by 50%.

A second enabling feature of the radar is that it operates in a slow-time multiple-input multiple-output (MIMO) mode. During operation, the radar sends coded pulses on an alternating basis through the two separate transmitting antennas strategically located at either end of the receiver array. On receiving, the pulses returned from both antennas are separated to form a virtual array that has an equivalent performance to a physical aperture of twice the length. As illustrated in Figure 3, in the vicinity of the indicated target, a nominal 3 dB improvement in the target-to-clutter ratio was realized. The signal-to-clutter improvements were gained from the reduction of the antenna azimuth beam width by one-half [8]. The azimuth sidelobes visible in the virtual aperture array (VAA) response curve were due to residual amplitude effects, due to the receiving of array cable losses. These effects can be mitigated with equalization techniques, which were not applied here. 

**Figure 4:**
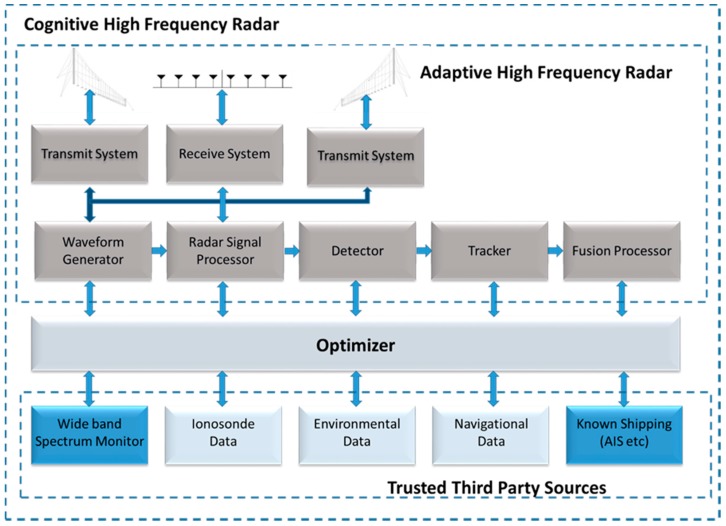
Canada’s third-generation cognitive HFSWR system consisting of an adaptive HFSWR (Act), the ingestion and processing of data into the optimizer (Sense), the determination of the optimum radar processing for the mission (Learn) and the application of optimized processing parameters (Decide).

As illustrated in Figure 4, the radar consists of three major sub-systems; an Adaptive, an Optimizer, and data from trusted third-party sources. For the demonstration system, these auxiliary data sources consisted of a wide-band spectrum monitoring system and a data fusion processor that associated the HF radar-derived surface tracks with the known shipping in the area obtained primarily from space-based interception of automatic identification system (AIS) reports. The data was combined to provide a comprehensive real-time overview of the surface vessel activity. 

The following sections present examples of the intelligent spectrum management that enables robust secondary-users’ operation whilst sharing the spectrum with primary communication users. The examples also illustrate how different waveform characteristics can be selected based on the sensed or known environment.

Examples of sense-and-adapt on receiving are presented, which illustrate how unwanted signals are minimized. A new adaptive detector is introduced, which minimizes false detections arising from clutter and other unwanted signals. This allows the threshold level within the detector to be lowered for improved radar sensitivity. Integrating the tracker within the radar architecture allows the system to move towards a track-before-detect mode by utilizing an adaptive, deferred decision, multi-target tracker based on the interacting multiple model (IMM) architecture that handles a high false plot rate without generating significant false tracks.

### 2.1. Intelligent Spectrum Utilization

HF Radars must operate on a NIB and NPB with respect to allocated users. This requires that that radar operates with frequency diversity, and that the system automatically selects the best frequency and bandwidth of operation based upon the local spectral measurements and limitations as specified by the radio license.

While operating at a specific frequency and bandwidth, the radar self-monitors for the presence of higher-priority licensed users. Simultaneously, a wideband spectrum monitoring system searches for unoccupied channels within the wider spectrum of operation. The spectrum management system generates a ranking of currently unoccupied channels, based on the mission and history of availability, using a fuzzy logic technique. On the detection of an unwanted signal within the radar operating channel, the spectrum management system instantaneously switches to the highest-ranked unoccupied channel. If no channels are available, the radar is configurable to either operate at a predefined default frequency or cease transmission until channels become available [9]. Using the fuzzy logic technique, the system implements the cognitive actions of perception, memory, judgment, and reasoning to determine the optimum frequency of operation. From the above, it can be seen that the cognitive radar utilizes both narrow-band and wideband channel data to support the actions of perception and memory. 

The availability of wideband spectrum data offers the opportunity to enhance the radar operation based on cognition. For example, impulses caused by distant lightning events, power lines or machinery result in the generation of impulsive noise. Impulses, by definition, are wideband spectral events. There are several competing factors within the narrow-band channel that challenge the detection and removal of impulse-contaminated data. The narrow-band channel is also occupied with clutter and target information. These signals typically have significant energy that can mask the noise floor elevation due to the impulse event. In addition, the pulse compression cycle in the HFSWR occurs over several pulse periods, and the removal of a corrupted pulse necessitates the removal of the entire pulse cycle. 

**Figure 5:**
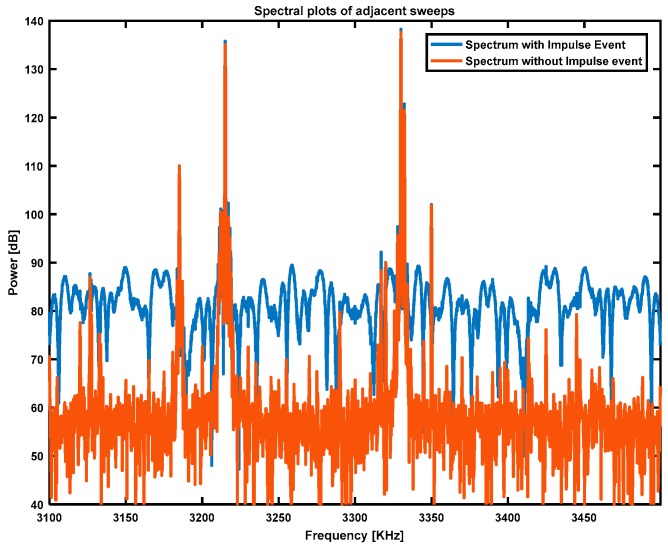
Illustration of the spectral power of two adjacent time sweeps. It can be readily observed that the sweep with the impulse event present (blue trace) had a considerably higher noise floor than the non-impulse-contaminated sweep (red trace).

The wideband channel, although occupied by many users, allows for the reliable detection of an impulse event by detecting noise floor elevation. As most users are restricted in bandwidth to a small fraction of the wideband channel, the noise floor increase is readily detected between occupied channels. To determine the noise floor and discern users from the noise floor, the time-sampled wideband data is converted to spectral power by the fast Fourier transform. The spectral power of two adjacent time sweeps is presented in Figure 5. It can be readily observed that the sweep with the impulse event present (blue trace) had a considerably higher noise floor than the non-impulse-contaminated sweep. This elevation in the noise floor is considerably harder to detect in the narrow-band channel with competing elements of clutter and target information. 

**Figure 6:**
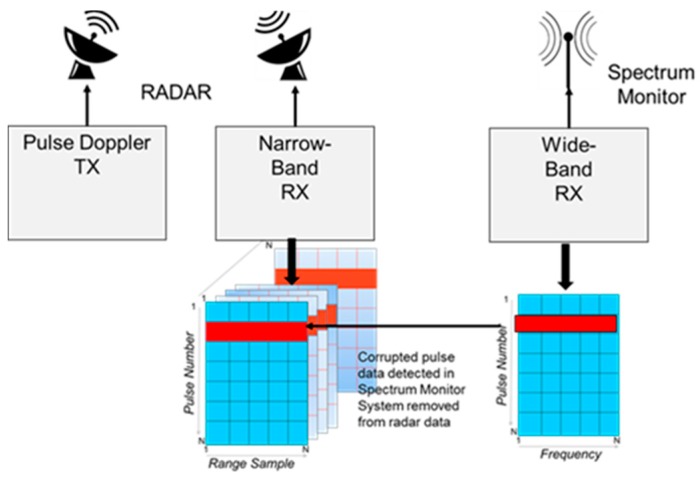
Wideband detection of impulse events with feedback to narrow-band target detection.

A simplified flow diagram for the detection and removal process described above is presented in Figure 6. Impulse events are detected using the wideband receiver. These events are then removed from the radar data and collected with the narrow-band receiver. Details of the approach are presented in [9]. 

### 2.2. Intelligence-Based Detection

This section considers adaptive variations of constant false alarm rate (CFAR) detectors [10,11,12]. HF radars operating in the 3–10 MHz region of the spectrum receive significant energy from reflections off the ionosphere. Due to the high reflectivity of the ionosphere, the received energy can be significantly larger than targets of interest. Thus, the ionospheric returns will generate a large quantity of CFAR detections, most prevalent along the edges of the returns. Standard CFAR techniques are ineffective in the non-homogenous environment with a large, abrupt change in power levels. With the long integration times necessary for the HF radar and the significant Doppler components of the ionosphere, the clutter edges smear significantly in the Doppler dimension so that the interference environment is non-homogenous. As the ionosphere is not a point target source, the azimuth component is diffuse, which leads to a significant quantity of target plots within the range, Doppler and azimuth space. The resulting false detections generate significant challenges to maximize the target detection and minimize the false track generation in these regions.

Advanced CFAR techniques can be successful in mitigating the quantity of false detections in non-homogenous environments. An adaptive CFAR process can be utilized to identify areas of high-clutter returns in the range–Doppler space to mitigate the false detections. Once the clutter edges have been mapped in the range–Doppler space, the CFAR parameters can be adjusted to decrease the probability of false detections on the edges. Simple techniques such as raising the threshold or adjusting the background test area dimensions are quite effective. 

The intelligence-based detector uses a hierarchy of CFAR algorithms to determine the optimal detector for a given environment and mission. An intelligence-based detector incorporates the knowledge learned from the environments into the decision process of the hybrid CFAR detection schemes. The knowledge gained is used in the selection of CFAR detectors, and is also used to adapt the CFAR parameters, including the reference window’s size, position and shape. To control the number of detections, the knowledge-aided detector adapts the detection threshold to higher levels in non-homogeneous environments, and to a lower percentage in homogeneous environments. A detector first senses the environments by applying a classifier. The classifier detects the areas of clutter and noise. With the knowledge gained from the local surroundings, the CFAR reference window adapts to best fit the distribution of the local noise/clutter environment. The CFAR sets the threshold so that the rate at which the false alarm occurs is constant. Hybrid CFAR schemes, including order statistics (OS)-CFAR, smallest of CFAR, cell-averaging (CA)-CFAR, and simplified censored CA-CFAR, can then be appropriately applied, based on the knowledge learned from the classifier of the local environment [13,14]. By deploying this advanced technique, the detector can adapt to the local clutter surroundings, and suppress the clutter breakthrough that can occur when operating in non-homogenous environments; meanwhile, the detector can adapt to providing a more realistic threshold estimation in homogenous environments, and perform robustly in the presence of multiple targets. As a result, the hierarchal detector performs optimally in both homogenous and non-homogenous environments, minimizing false nuisance detections, and increasing the probability of detection for targets. 

**Figure 7:**
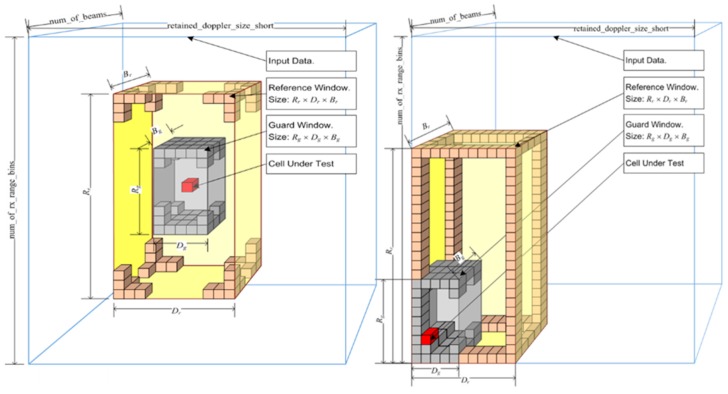
Construct of the CFAR engine.

As illustrated in Figure 7, the intelligence-based detector is constructed to adapt to specific range, Doppler and beam areas of the detection region. Typically, OS-CFAR types are used for clutter regions, and CA-CFAR, for noise-limited regions. The algorithm handles edge effects by freezing the evaluation area at the edges of clutter, and moving the test cell from within the evaluation area. This allows for the detection of targets that are adjacent to strong clutter. This technique produces a more accurate estimate of interference levels, compared to standard techniques that include the strong clutter into the test cells as well as clutter boundaries in the reference window, thereby biasing the detection statistics of the region.

Adaptive CFAR techniques minimize the quantity of false detections in non-homogenous environments. The adaptive CFAR process identifies areas in the range–Doppler space to mitigate the false detection problem. Once the clutter edges have been mapped in the range–Doppler space, the CFAR parameters are adjusted to decrease the probability of false detections on the edges. Simple techniques such as raising the threshold or adjusting the background test area dimensions are quite effective. In our solution, we have extended the two-clutter model in [15] to a three-clutter-type model, to identify areas of low-, medium- and high-clutter for each range–Doppler plane of each azimuth beam. Within each of these zones, the CFAR parameters are specified, with the overall goal to maximize the target detection probabilities in the low- and medium-clutter areas, and minimize false detections in the high-clutter regions. 

**Figure 8:**
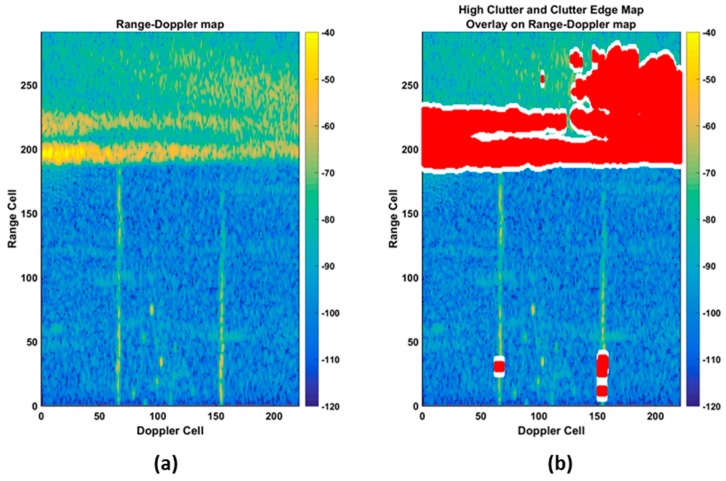
(**a**) Typical range–Doppler plane, with (**b**) high-clutter areas, and edges identified with a red and white overlay.

**Figure 9:**
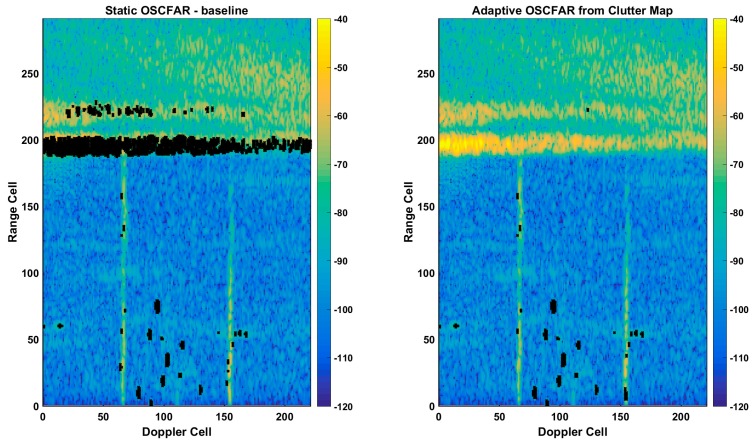
Range–Doppler plane with baseline CFAR detection overlay (**left**) and adaptive CFAR detections overlay (**right**) showing an approximately 90% reduction in CFAR detection with adaptive CFAR.

Figure 8a presents a typical range–Doppler plane mapped to color (power), whereas Figure 8b shows the same data with the clutter areas and clutter edges overlaid with a red and white overlay. Within this area, the CFAR parameters are adapted to minimize the detection likelihood due to clutter edges. A comparison of the performance between a non-adaptive CFAR and our adaptive CFAR is shown in Figure 9. It can be observed that a balanced detection process was achieved with maximum sensitivity in non-clutter (homogenous) regions and a reduced sensitivity at the clutter edge. In the example presented, our adaptive CFAR decreased the total CFAR count by approximately 90%, while maintaining valid detections on all targets. The dramatic CFAR-hit reduction results for significantly reduced processing loads on the tracker allowed for more complex tracking techniques to be utilized, resulting in the generation of less false tracks and a more consistent tracking of legitimate targets. 

### 2.3. Intelligence-Based Tracking

A general overview of the tracker used within the radar system can be found in [16]. The application of cognition introduces the use of external knowledge and intelligence into the tracking process. Intelligence-based tracking is used to maximize the probability of a wanted track, whilst minimizing the probability of an unwanted or otherwise erroneous track.

- Intelligence based tracking can be implemented using one or all the following techniques:
- Feature aided tracker (FAT)
- Special track processing techniques
- Enhanced tracking filters
- Enhanced track classification algorithms

FAT is an emerging technology that has been specifically designed for challenging tracking scenarios, e.g., the tracking of weakly detected targets in heavy clutter. FAT begins with the identification of existing and new target features. A feature extraction algorithm is then designed to extract features from a continuous string of signature signals that are commensurate with the targets of interest being tracked over time. Potentially discriminating target features are power, radar cross section (RCS) or the size of the target, for instance. Kinematic and target features are then jointly exploited in a probabilistic manner for simultaneous tracking and identification or classification. Target features and the kinematic properties of the target (in track) jointly determine the weights for data association, which not only reduces miscorrelation, but as a result, greatly improves track continuity. Thus, in demanding tracking scenarios, FAT renders a significant improvement over conventional tracking schemes that are based solely on the kinematic properties of the target in track. Examples of the application of FAT techniques can be found in [17,18].

To further improve performance, the tracker also incorporates special processing techniques to reduce the occurrence of false tracks and seduced tracks in a high-clutter environment. Some of these techniques are described below.

### 2.4. Adaptive Track Promotion Logic

Adaptive track promotion logic defines a track lifeline as potential, tentative, confirmed and deleted. Details of the algorithm can be found in [16]. As an example, a typical fixed-track promotion logic may be defined as 3-4-6. This logic states that a tentative track (that is newly formed with two plots) will be deleted if it has three consecutive misses. A tentative track will be promoted to a confirmed track if there are four accumulated associations. If a confirmed track fails to associate plots in six consecutive updates (i.e., six consecutive misses), it will be deleted. For comprehensiveness, the logic can be written as 2-3-4-6, where the number 2 is the number of misses to delete a potential track. However, the number 2 is never changed (in our scheme); thus, the more concise form of 3-4-6 is typically adopted to describe the track promotion logic. 

A fixed track promotion logic is adequate for a homogeneous environment, but less so for a heterogeneous environment. For instance, in a “clean” environment, it would be prudent to select a promotion logic that resulted in a timely track confirmation. However, in an environment of heavy clutter, the higher probability of a false track may preclude such a configuration for the track promotion logic. Thus, it is desirable to employ an adaptive promotion logic that is not fixed, but varies according to the local environment.

When adaptive promotion logic is utilized, the track promotion logic is adjusted based on the clutter distribution. Adaptive track promotion logic relies on a plot concentration map to monitor the plot activity within the instrumented range of the radar, and to identify areas with excessive plot activity. In areas of excessive plot activity, the likelihood of false track confirmation from a sequence of extraneous plots is high; thus, the parameters governing the track promotion are locally adapted within these areas. This adaptation significantly reduces the rate of confirming false tentative tracks in areas of excessive clutter. 

Other parameters, such as the gate threshold for ellipsoidal gating, may also be adapted, and the adaptation may even be augmented by knowledge of the local infrastructure or terrain, such as in map-aided processing. 

**Figure 10:**
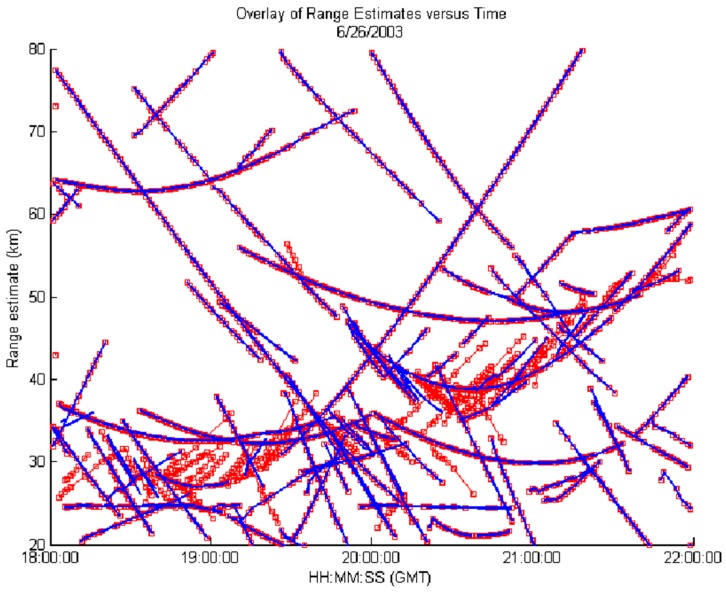
Range vs. time track overlay comparing fixed (red) and adaptive (blue) promotion logic tracking.

Figure 10 shows range vs. time track data between a tracker employing fixed promotion logic (red tracks) and a tracker using adaptive promotion logic (blue tracks). Raw input plot data was collected from an operational HFSWR system. Across 4 hours of data, it is apparent that the tracker using a fixed promotion logic generated significantly more false tracks (at near-range) than the tracker which adapted its promotion logic. Adaptive promotion logic dynamically adapts the gate threshold and the parameters governing track promotion according to local clutter conditions, and consequently, renders a track picture that is effectively free of false tracks, while demonstrating negligible impacts on the tracking of genuine targets. As shown above, adaptive promotion logic provides an effective mechanism to mitigate false tracks due to strong clutter with non-stationary statistics, and thus, it has been implemented in the tracker for the third-generation HFSWR system.

### 2.5. Map-Aided Processing

A map of the terrain or local infrastructure can also be combined with the plot concentration map to quickly identify suspect plots/tracks. For example, knowledge of the coastline could be used to suppress land-based tracks, whereas knowledge about the local infrastructure could allow the tracker to quickly identify false plots/tracks caused by active offshore structures. This suppression can also be used in radar spectra. Map-aided processing leverages knowledge about the local surroundings, and can dramatically reduce the rate of false tracks caused by clutter.

Intelligence-based tracking addresses the decision problems related to track confirmation and track termination. In general, such decisions will be a function of the probability of target detection, local false alarm density, sequence of associated plots, and the lifetime of the track in question. For a better utilization of all the available information, track confidence can also be used to establish/terminate a track. Track confidence will typically require sensible estimates for the local false alarm density and the ever-changing target detection probability. Another simpler approach would be to merely assign a higher priority to longer, more consistent tracks. The rationale is that by assigning a higher priority to longer, more consistent tracks, the likelihood that a real target plot will be associated with a false track will tend to be reduced. This technique termed “processing prioritization” promotes track continuity for established tracks. Established tracks are maintained through a tracking filter, as discussed in the following section.

### 2.6. Enhanced Tracking Filters

There is a plethora of tracking filters, the most common (in practice) being the alpha−beta filter and the Kalman filter. These single-model filters cannot provide a sensible compromise between two conflicting requirements: significant noise reduction during uniform motion, and a fast response during a maneuver. Consequently, multiple model filters were considered to better handle the tracking of maneuvering targets. A notable development is the IMM filter, which has emerged as the most cost-effective approach for the tracking of maneuvering targets via multiple model filtering. This approach is very efficient when handling a large-scale target situation consisting of many maneuvering targets, and it has been shown to outperform both the alpha−beta and the Kalman filters in such an environment [19].

### 2.7. Enhanced Track Classification Algorithms

This has been implemented through a classifier-aided tracker based on the support vector machine (SVM) technique for target track classification [20]. By performing plot/track classification, the classifier aids the tracker in selecting the appropriate plot to track assignment. The classifier-aided tracker, as shown in [18], yields considerable improvements in the presence of high-clutter sources, with respect to conventional tracking techniques.

## 3. Results

**Figure 11:**
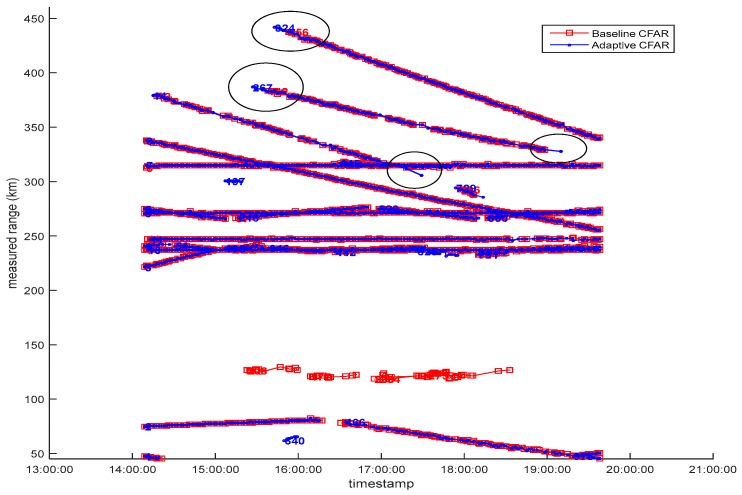
Overlay of range vs. time track picture via baseline and adaptive CFAR detectors. It can be observed that the adaptive CFAR detector extended the track life and initiated tracks earlier (circles above a range of 250 km), and that a number of false tracks arising from ionospheric clutter or other sources (at 120 to 140 km) were completely eliminated from the track picture.

As discussed previously, the use of an intelligence-based CFAR detector can result in a significant reduction in the number of false detections arising from the presence of unwanted clutter. This reduces the processing load on the tracker, and allows more resources to be allocated to advanced tracking techniques, with the result of maximizing the probability of tracking, whilst minimizing the probability of false tracks. As an example, Figure 11 presents a range vs. time track picture overlay for a 6 hour recording based on raw radar data collected from a HFSWR system located near Halifax, Nova Scotia. The red tracks were obtained through the baseline CFAR detector whereas the blue tracks were obtained through the adaptive CFAR detector. The baseline detector employed the same threshold and reference window for each test cell, whereas the adaptive CFAR adjusted the parameterization (e.g., threshold and background test area) according to whether the test cell resided in an area of low-, medium- or high-clutter. Both sets of data used the same tracker. Owing to the adaptive CFAR detector, it can be seen that several long-range targets enjoyed an extended track life and earlier detection (circles above a range of 250 km), and that a number of false tracks arising from ionospheric clutter were completely eliminated from the track picture (near a range of 120 km).

## 4. Conclusions

This paper presented an overview of Canada’s third-generation HFSWR that has been developed to form the foundation layer of a maritime domain awareness system to provide enforcement agencies with real-time persistent surveillance out to and beyond the 200 nautical mile EEZ. It has been shown that cognitive sense-and-adapt technology and dynamic spectrum management ensure robust and resilient operation within the highly congested HF band. It has been shown that dynamic spectrum access enables the system to operate on a NIB and NPB without impacting other spectrum users. The paper has discussed adaptive signal processing techniques that mitigate against electrical noise, interference and clutter. Sense-and-adapt techniques applied at the CFAR detector and tracker stages have been shown to dramatically reduce the number of false detections associated with clutter, and thereby significantly reduce the processing load on the tracker. This allows more advance tracking techniques to be deployed, such that the system maximizes the probability of tracking, whilst minimizing the probability of false or otherwise erroneous track data.
